# Cerebral vascular function following the acute consumption of caffeinated artificially- and sugar sweetened soft drinks in healthy adults

**DOI:** 10.3389/fnhum.2022.1063273

**Published:** 2022-12-22

**Authors:** Emma L. Reed, Morgan L. Worley, Paul J. Kueck, Leonard D. Pietrafasa, Zachary J. Schlader, Blair D. Johnson

**Affiliations:** ^1^Human Integrative Physiology Lab, Department of Exercise and Nutrition Sciences, University at Buffalo, Buffalo, NY, United States; ^2^H.H. Morris Human Performance Laboratories, Department of Kinesiology, Indiana University, Bloomington, IN, United States

**Keywords:** autoregulation, reactivity, cerebral blood velocity, cerebral vascular conductance, blood pressure, sweetened beverage

## Abstract

Chronic consumption of sugar- and artificially-sweetened beverages (SSB and ASB) are associated with an increased risk of stroke but it is unclear how acute consumption influences cerebral vascular function.

**Purpose:** We hypothesized that: (1) acute consumption of SSB and ASB would augment dynamic cerebral autoregulation (dCA) and attenuate cerebral vascular reactivity to hypercapnia (CVR_CO2_) compared to water; and (2) dCA and CVR_CO2_ would be attenuated with SSB compared to ASB and water.

**Methods:** Twelve healthy adults (age: 23 ± 2 years, four females) completed three randomized trials where they drank 500 ml of water, SSB (Mountain Dew^®^), or ASB (Diet Mountain Dew^®^). We measured mean arterial pressure (MAP), middle and posterior cerebral artery blood velocities (MCAv and PCAv), and end-tidal CO_2_ tension (PETCO_2_). Cerebral vascular conductance was calculated as cerebral artery blood velocity/MAP (MCAc and PCAc). Twenty min after consumption, participants completed a 5 min baseline, and in a counterbalanced order, a CVR_CO2_ test (3%, 5%, and 7% CO_2_ in 3 min stages) and a dCA test (squat-stand tests at 0.10 Hz and 0.05 Hz for 5 min each) separated by 10 min. CVR_CO2_ was calculated as the slope of the linear regression lines of MCAv and PCAv vs. PETCO_2_. dCA was assessed in the MCA using transfer function analysis. Coherence, gain, and phase were determined in the low frequency (LF; 0.07–0.2 Hz) and very low frequency (VLF; 0.02–0.07 Hz).

**Results:** MCAv and MCAc were lower after SSB (54.11 ± 12.28 cm/s, 0.58 ± 0.15 cm/s/mmHg) and ASB (51.07 ± 9.35 cm/s, 0.52 ± 1.0 cm/s/mmHg) vs. water (62.73 ± 12.96 cm/s, 0.67 ± 0.11 cm/s/mmHg; all *P* < 0.035), respectively. PCAc was also lower with the ASB compared to water (*P* = 0.007). MCA CVR_CO2_ was lower following ASB (1.55 ± 0.38 cm/s/mmHg) vs. water (2.00 ± 0.57 cm/s/mmHg; *P* = 0.011) but not after SSB (1.90 ± 0.67 cm/s/mmHg; *P* = 0.593). PCA CVR_CO2_ did not differ between beverages (*P* > 0.853). There were no differences between beverages for coherence (*P* ≥ 0.295), gain (*P* ≥ 0.058), or phase (*P* ≥ 0.084) for either frequency.

**Discussion:** Acute consumption of caffeinated SSB and ASB resulted in lower intracranial artery blood velocity and conductance but had a minimal effect on cerebral vascular function as only MCA CVR_CO2_ was altered with the ASB compared to water.

## 1 Introduction

In the United States, 63% of adults self-reported consuming sugar-sweetened beverages (SSB) daily (Chevinsky et al., 2021). Chronic consumption of SSB is associated with an increased risk of hypertension (Kim and Je, 2016; Malik and Hu, 2019), type 2 diabetes (Imamura et al., 2015), and stroke (Bernstein et al., 2012; Narain et al., 2016; Pacheco et al., 2020). Moreover, many individuals consume artificially sweetened beverages (ASB) to lower caloric intake and for disease management (Fitch and Keim, 2012; Gardner et al., 2012). As such, approximately 20% of adults in the United States consume ASB daily (Fakhouri et al., 2012). However, daily consumption of ASB has been associated with an increased risk of stroke and dementia (Pase et al., 2017). Additionally, caffeine is common in SSB, ASB, and other beverages such as coffee and tea (Chou and Bell, 2007; Reyes and Cornelis, 2018). Regular consumption of caffeinated coffee is associated with a reduced risk of stroke (Larsson and Orsini, 2011). Thus, it is likely the ingredients that are unique to caffeinated coffee reduce stroke risk. Nevertheless, the consumption of SSB or ASB is associated with an increased risk of diseases associated with poor cerebral vascular function but very little is known about the acute cerebral vascular effects following the ingestion of these beverages.

The most common sweeteners in SSB are high-fructose corn syrup (HFCS; ~60% fructose, ~40% glucose) or sucrose (50% fructose, 50% glucose). HFCS consumption increases free fructose in the circulation which might contribute to the negative health outcomes associated with HFCS consumption (Le et al., 2012). The most common sweeteners in ASB are sucralose and aspartame. There is some evidence that these constituents of SSB and ASB can acutely affect hemodynamics and/or peripheral vascular function. The acute consumption of an HFCS beverage, but not a sucrose-sweetened beverage, elevated peak post-consumption systolic blood pressure, renal vascular resistance, and serum uric acid (Le et al., 2012; Chapman et al., 2020). The acute consumption of fructose alone results in elevated blood pressure and plasma levels of norepinephrine in humans (Jansen et al., 1987; Brown et al., 2008; Grasser et al., 2014). Additionally, there is a reduction in endothelial nitric oxide synthase expression in aortic endothelial cells exposed to fructose (Glushakova et al., 2008). Whereas, oral consumption, intraduodenal infusions, or cultured vascular cells exposed to artificial sweeteners, such as sucralose and aspartame, do not appear to acutely influence blood pressure or endothelial cell function (Memon et al., 2014; Pham et al., 2018; Schiano et al., 2020). However, there is evidence that 10 weeks of consuming artificial sweeteners (sucralose and acesulfame potassium) reduces acetylcholine-induced endothelium-dependent vasodilation in rodent aortas (Risdon et al., 2020). Caffeine is also a common ingredient in SSB and ASB that might influence cerebral vascular function. Caffeine is an adenosine receptor antagonist and inhibits vasodilation (Cameron et al., 1990; Coney and Marshall, 1998; Phillis and O’Regan, 2003). Specific to the cerebral vasculature, coffee ingestion reduces intracranial artery blood velocity and improves cerebral autoregulation (Sasaki et al., 2016). Although there is evidence for changes in peripheral vasculature with acute consumption, it is still unclear whether acute consumption of caffeinated beverages with different sweeteners (HFCS and artificial sweeteners) influences cerebral vascular function.

Cerebral vascular function, quantified by dynamic cerebral autoregulation and cerebral vascular reactivity, is vital to maintain cerebral blood flow during changes in arterial blood pressure, arterial blood gases, local metabolism, and alterations in autonomic nervous system activation (Willie et al., 2014). Arterial blood pressure fluctuations can occur during common activities of daily living such as moving from a seated to a standing position. Inadequate maintenance of cerebral blood flow during postural changes can result in light-headedness, dizziness, and in some cases syncope (e.g., orthostatic hypotension). Cerebral autoregulation and cerebral vascular reactivity to carbon dioxide are both modulated by local vascular factors (i.e., nitric oxide), myogenic responses, and autonomic innervation (Schmetterer et al., 1997; Zhang et al., 2002; Toda et al., 2009; Hamner et al., 2010). However, it is unknown if the acute consumption of SSB and/or ASB influence cerebral vascular function and the maintenance of cerebral blood flow during orthostatic challenges. Along these lines, cerebral vascular reactivity is the cerebral blood flow/velocity response to changes in the partial pressure of arterial blood gases, such as carbon dioxide (Kety and Schmidt, 1948; Hoiland et al., 2019) but it is currently not known how cerebral vascular reactivity is affected by the acute consumption of SSB and ASB.

Therefore, we investigated dynamic cerebral autoregulation and cerebral vascular reactivity to hypercapnia in healthy adults following the acute consumption of an SSB, an ASB, and water. Our first aim was to investigate the effects of commercially available caffeinated sugar- and artificially-sweetened soft drinks on cerebral vascular function. We hypothesized that the acute consumption of SSB and ASB would augment dynamic cerebral autoregulation and attenuate cerebral vascular reactivity to hypercapnia compared to water due to the effects of caffeine. Our second aim was to examine the effects of different sweeteners in commercially available caffeinated soft drinks on cerebral vascular function. Because our SSB contained HFCS, we hypothesized that the acute consumption of an SSB would attenuate dynamic cerebral autoregulation and cerebral vascular reactivity to hypercapnia compared to acute consumption of an ASB and water.

## 2 Materials and Methods

Twelve participants (age: 23 ± 2 years, height: 171 ± 10 cm, weight: 72 ± 11 kg; body mass index: 24 ± 3 kg/m^2^; four females) volunteered for the study. All participants self-reported to be free of cardiovascular, respiratory, metabolic, and neurological disorders and were regularly physically active. Female participants were tested within 10 days of the start of their self-reported menstrual cycle and were not pregnant (confirmed by a urine pregnancy test at the beginning of each study visit). All study visits were scheduled with at least 24 h between visits and started within the same hour of the day to account for any potential circadian variations. The study involving human participants was reviewed and approved by the Institutional Review Board of the University at Buffalo. The participants provided written informed consent to participate in the study. The study was performed in accordance with the latest revision of the Declaration of Helsinki with the omission of registration in a database.

### 2.1 Experimental design

**Figure 1:**
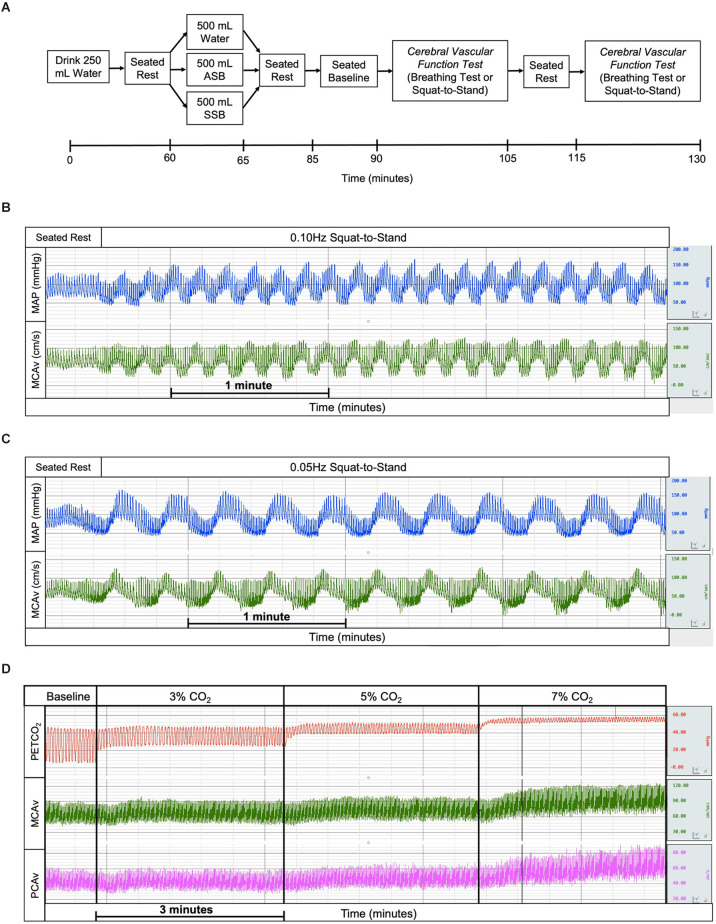
Experimental study visits timeline **(A)** where the experimental drinks were randomized and the order of cerebral vascular function tests was randomized and counterbalanced. The example tracings of mean arterial pressure (blue), middle cerebral artery blood velocity (green), end-tidal carbon dioxide (red), and posterior cerebral artery blood velocity (magenta) for squat-to-stand at 0.10 Hz **(B)**, squat-to-stand at 0.05 Hz **(C)**, and cerebral vascular reactivity to hypercapnia **(D)** tests. ASB, artificially-sweetened beverage; SSB, sugar-sweetened beverage.

Participants completed three study visits that were identical with the exception of the beverage that was consumed (Figure 1A). During the visits, participants consumed 500 ml (16 oz) of the experimental beverage: water, an SSB (Mountain Dew^®^, PepsiCo, Purchase, NY), or an ASB (Diet Mountain Dew^®^, PepsiCo, Purchase, NY) beverage in a randomized study design. This volume for the experimental beverages was chosen as 500 ml (16 oz) is the size of one standard bottle of commercially available soft drinks. Participants and researchers were blinded to the soft-drink beverages, however, it was not possible to blind participants to the water condition due to the lack of flavor. To avoid any potential effect of carbonation, the soft drinks were decarbonated 48 h prior to consumption by being stored in a wide-mouth bottle loosely covered to prevent contamination. Each study visit had two different tests to assess cerebrovascular function; a three-stage hypercapnic breathing test to assess cerebral vascular reactivity to hypercapnia and squat-to-stand tests performed at two different frequencies to assess dynamic cerebral autoregulation. The order of the tests within the study visits was counterbalanced among the participants. We selected Mountain Dew^®^ as the SSB because it has one of the highest sugar contents for beverages sweetened with HFCS (~36 g of fructose and ~25 g of glucose; Walker et al., 2014) of commercially available soft drinks. We selected ASB Mountain Dew^®^ as the ASB to match for flavor and caffeine content (76 mg). In our prior work, we identified that the acute consumption of these beverages influences the renal vasculature (Chapman et al., 2020).

### 2.2 Experimental protocol

Participants arrived at the temperature-controlled laboratory (water: 24 ± 2°C, 30 ± 6% relative humidity; SSB: 24 ± 2°C, 29 ± 9% relative humidity; ASB: 24 ± 3°C, 27 ± 5% relative humidity) having refrained from exercise, alcohol, and caffeine for 12 h, and food for 2 h. Participants voided their bladders to provide a urine sample to confirm euhydration (urine specific gravity < 1.020; American College of Sports et al., 2007), and nude body weight was measured in a private room. Participants consumed 250 ml of water and rested in a seated position for 60 min during instrumentation. After this period, participants voided their bladders and then consumed 500 ml of the experimental beverage (SSB, ASB, or water) within a 5-min period. Participants remained seated for an additional 20 min after beverage consumption. This was followed by a 5-min baseline, the first cerebrovascular function test (cerebral vascular reactivity to hypercapnia or dynamic cerebral autoregulation), 10 min of quiet rest, and finally the second cerebrovascular function test (cerebral vascular reactivity to hypercapnia or dynamic cerebral autoregulation). Dynamic cerebral autoregulation (dCA) was assessed using two 5 min squat-to-stand tests at 0.10 Hz (Figure 1B) and 0.05 Hz (Figure 1C) to induce oscillatory fluctuations in blood pressure (Birch et al., 1995; Zhang et al., 1998; Claassen et al., 2009; Smirl et al., 2015) with a 5 min break between squat-to-stand tests. Participants were instructed to squat and stand to a metronome and squat to the level of a chair without touching the chair to ensure consistent squat depth within and between trials. The cerebral vascular reactivity to hypercapnia (CVR_CO2_) test was performed using 3-min stages breathing 3% CO_2_, 5% CO_2_, and 7% CO_2_ (all gases contained 21% O_2_ balanced with N_2_; Figure 1D).

### 2.3 Instrumentation and measurements

Height and body weight were measured with a stadiometer and electronic scale (Sartorius, Bohemia, NY). Urine specific gravity (USG) was measured with a refractometer (Atago, Bellevue, WA). Heart rate was continuously measured *via* a 3-lead electrocardiogram (250 Hz; DAC100C, Biopac Systems, Goleta, CA). Beat-to-beat mean arterial pressure was measured *via* the Penaz method (62.5 Hz; Finometer Pro, FMS, Amsterdam, Netherlands) and intermittently confirmed *via* electrosphygmomanometry of the brachial artery (Tango M2; SunTech, Raleigh, NC). From the blood pressure waveform, stroke volume was estimated *via* Modelflow (Wesseling et al., 1993). Cerebral blood velocities of the left middle cerebral artery (MCAv) and right posterior cerebral artery (PCAv) were measured simultaneously using 2 MHz transcranial Doppler ultrasound probes (1 kHz; DWL Compumedics, Germany) through the left and right temporal windows, respectively. The depth and gain for both intracranial arteries were recorded at the first study visit and these settings were used for the remaining study visits. The partial pressure of end-tidal carbon dioxide tension (PETCO_2_) was measured with capnography (62.5 Hz; Nonin Medical, Plymouth, MN).

### 2.4 Data and statistical analyses

All data were sampled continuously *via* a data acquisition system (Biopac MP150, Goleta, CA). The mean data of the last 2 min of the 5-min rest period were used as the post-consumption baseline values. dCA was assessed using transfer function analysis (WinCPRS, Absolute Aliens Oy, Turku, Finland) of the beat-to-beat blood pressure (input signal) and the MCAv (output signal) in two frequency domains (Zhang et al., 1998) during each squat-to-stand frequency. The posterior cerebral artery was not analyzed due to the number of data points that had to be excluded due to some noise in the waveforms that made transfer function analysis uninterpretable (Claassen et al., 2016). The 0.10 Hz squat-to-stand was analyzed in the LF (0.07–0.2 Hz) and the 0.05 Hz squat-to-stand was analyzed in the VLF (0.02–0.07 Hz) to match the operant ranges of cerebral autoregulation (Claassen et al., 2009). Transfer gain reflects the magnitude and phase reflects the time delay between arterial blood pressure (input signal) and cerebral blood velocity (output signal). Negative phase values were excluded in both frequency bands due to the “wrap around” effect. Coherence values reflect the linearity between arterial blood pressure and cerebral blood velocity where values closer to 1 represent a more linear relation. An improvement in cerebral autoregulation is marked by a decrease in gain and an increase in phase indicating a buffering of arterial blood pressure in the cerebral vasculature (Claassen et al., 2016). The mean of the 5-min squat-to-stand protocol for both frequencies for each physiological variable was calculated and shown as mean ± standard deviation (Table 2 and Figure 4). CVR_CO2_ in the MCA and PCA were calculated as the slope of the linear regression analysis of peak PETCO_2_ to the average MCAv or PCAv (Xie et al., 2006), respectively, for every 30 s of the breathing test for all three %CO_2_ (Barnes et al., 2012, 2013). CVR_CO2_ is used as an index of cerebral nitric oxide bioavailability. However, nitric oxide is not the only endothelial-derived response that contributes to the increases in blood flow during hypercapnia (Iadecola and Zhang, 1994; Schmetterer et al., 1997). Hyperpolarization of endothelial cells due to the activation of voltage-gated potassium channels also contributes to vascular relaxation thus increasing blood flow (Kitazono et al., 1995; Nelson and Quayle, 1995). The mean of the 30 s intervals for each 3-min stage of the hypercapnia breathing test for each physiological variable was calculated (Table 2 and Figure 6). Cardiac output was calculated as the product of stroke volume and heart rate and total peripheral resistance was calculated as the quotient of mean arterial pressure and cardiac output. Cerebral vascular conductance, an indirect quantification of arterial vasomotor tone, was calculated as the quotient of cerebral artery blood velocity and mean arterial pressure for both the MCA and PCA.

Data were analyzed using Prism software (Version 9, GraphPad Software, La Jolla, CA). Post-consumption baseline, dynamic cerebral autoregulation, and cerebral vascular reactivity values were analyzed using one-way mixed-effects models. Baseline values during the cerebral vascular reactivity test were analyzed with a two-way mixed-effects model for time (inhaled %CO_2_) × condition (drink). If a significant interaction or main effect was found, we used the *post-hoc* Holm-Sidak multiple comparisons test to determine where differences existed, and *P*-values are reported where possible. Data are reported as mean ± standard deviation and comparisons were considered significantly different if *P* < 0.050.

**Table 1:** Seated baseline cardiovascular and end-tidal carbon dioxide values 20 min after consuming each experimental beverage.

	Post-Consumption Baseline				
Variable	Water	ASB	SSB	Mixed Effects Model *P*-value
Heart Rate (bpm)	64 ± 7	65 ± 8	67 ± 9^W^	0.039
Mean arterial pressure (mmHg)	95 ± 13	99 ± 12	95 ± 11	0.298
Systolic blood pressure (mmHg)	128 ± 18	131 ± 14	131 ± 15	0.702
Diastolic blood pressure (mmHg)	72 ± 11	77 ± 11	77 ± 9	0.253
Stroke volume (ml)	89 ± 19^∧^	82 ± 18	93 ± 12	0.195
Cardiac output (L/min)	5.7 ± 1.4^∧^	5.3 ± 1.5	6.2 ± 1.0	0.129
Total peripheral resistance (mmHg/L/min)	16.7 ± 5.4^∧^	18.6 ± 5.8^∧^	15.6 ± 2.7	0.204
End-tidal carbon dioxide (mmHg)	44 ± 3	42 ± 2	43 ± 2	0.037

*Data are reported as mean ± standard deviation (*n* = 12, 4 females, ^∧^*n* = 11, 3 females). Mixed effects models were used to compare data between conditions. If a mixed effects model revealed a significant effect a *post-hoc* Holm-Sidak multiple comparisons test was used to determine where differences existed. ^W^Different from Water (*p* < 0.050); ASB, artificially-sweetened beverage; SSB, sugar-sweetened beverage*.

**Table 2:** Cardiovascular responses during each stage of the cerebral vascular reactivity to hypercapnia test and each squat-to-stand frequency of the dynamic cerebral autoregulation tests.

Test	Variable	Stage	Water	ASB	SSB	Time	Drink	Time × Drink
**Cerebral Vascular Reactivity to Hypercapnia (CVR_CO2_)**	**Heart Rate (bpm)**	3% CO_2_	67 ± 9^B^	68 ± 9^B^	72 ± 8^WS^	<0.001	0.009	0.083
		5% CO_2_	70 ± 9^B^	71 ± 10^B^	76 ± 8^BWS^			
		7% CO_2_	75 ± 10^B^	73 ± 10^B^	78 ± 9^BS^			
	**Stroke Volume (ml)**	3% CO_2_	88 ± 23	86 ± 17	91 ± 11	0.246	0.518	0.284
		5% CO_2_	90 ± 24	88 ± 17	91 ± 11			
		7% CO_2_	96 ± 23	89 ± 17	94 ± 11			
	**Cardiac Output (L/min)**	3% CO_2_	6.1 ± 2.0	5.8 ± 1.4^B^	6.6 ± 1.1	0.012	0.208	0.294
		5% CO_2_	6.6 ± 2.2^B^	6.2 ± 1.3^B^	7.0 ± 1.3^B^			
		7% CO_2_	7.4 ± 2.4^B^	6.5 ± 1.5^B^	7.3 ± 1.3^B^			
	**Total Peripheral Resistance (mmHg/L/min)**	3% CO_2_	16.7 ± 5.5	18.7 ± 6.90	15.1 ± 2.9	0.167	0.119	0.475
		5% CO_2_	16.2 ± 5.5^B^	17.9 ± 6.3	14.9 ± 2.9			
		7% CO_2_	14.9 ± 5.4^B^	17.8 ± 6.3	15.0 ± 3.1			
**Dynamic Cerebral Autoregulation (dCA)**	**Heart Rate (bpm)**	0.10 Hz	82 ± 7^B^	82 ± 7^B^	86 ± 7^BWS^	-	0.006	-
		0.05 Hz	82 ± 7^B^	84 ± 8^B^	87 ± 7^BS^	-	0.012	-
	**Stroke Volume (ml)**	0.10 Hz	109 ± 19^B^	97 ± 15^B^	105 ± 15^B^	-	0.094	-
		0.05 Hz	100 ± 15	92 ± 18^B^	98 ± 11^B^	-	0.272	-
	**Cardiac Output (L/min)**	0.10 Hz	9.1 ± 8.0^B^	7.0 ± 8.8^B^	8.1 ± 9.9^BS^	-	0.031	-
		0.05 Hz	7.7 ± 1.7^B^	7.6 ± 1.7^B^	8.5 ± 1.9^BS^	-	0.152	-
	**Total Peripheral Resistance (mmHg/L/min)**	0.10 Hz	11.3 ± 2.7^B^	13.7 ± 3.9^B^	11.8 ± 2.5^B^	-	0.069	-
		0.05 Hz	12.1 ± 2.5^B^	14.7 ± 5.6^B^	11.8 ± 1.9^B^	-	0.084	-

*Data obtained from the cerebral vascular reactivity to the hypercapnia test were compared using mixed effects models. Data acquired during dynamic cerebral autoregulation tests were compared using mixed effects models for each frequency. Mixed effects models were used to compare data between conditions. If a mixed effects model revealed a significant effect, a *post-hoc* Holm-Sidak multiple comparisons test was used to determine where differences existed. Values are reported as mean ± standard deviation. ^B^Different from baseline (*P* < 0.05); ^W^Different from Water (*P* < 0.050); ^S^Different between soft drinks (*P* < 0.050); ASB, artificially-sweetened beverage; SSB, sugar-sweetened beverage*.

## 3 Results

**Figure 2:**
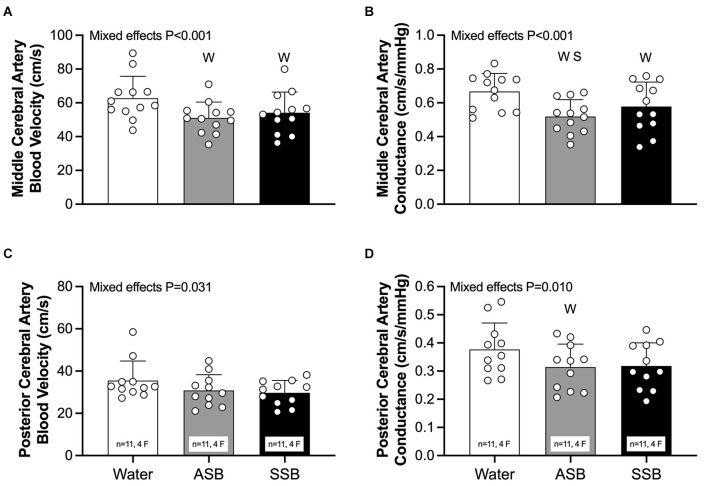
Seated baseline values 20 min after consuming each experimental beverage for middle cerebral artery blood velocity **(A)**, middle cerebral artery vascular conductance **(B)**, posterior cerebral artery blood velocity **(C)**, and posterior cerebral artery vascular conductance **(D)**. Data are reported as mean ± standard deviation. Mixed effects models were used to compare data between conditions. If a mixed effects model revealed a significant effect, a *post-hoc* Holm-Sidak multiple comparisons test was used to determine where differences existed. *n* = 12, four females unless otherwise noted. Missing data occurred due to the loss of signal and/or inability to obtain a quality signal while maintaining the same depth and/or gain with the transcranial Doppler signal. ^W^Different from Water (*P* < 0.050); ^S^Different between soft drinks (*P* < 0.050); ASB, artificially-sweetened beverage; SSB, sugar-sweetened beverage.

There were no differences (*P* = 0.798) in pre-consumption USG among the water (1.009 ± 0.007), SSB (1.008 ± 0.006), and ASB (1.008 ± 0.006) study visits. At the post-consumption baseline (Table 1), heart rate was higher after SSB (*P* = 0.047) compared to water. MCAv (Figure 2A; both *P* < 0.002) and MCAc (Figure 2B; *P* < 0.041) were lower after SSB and ASB compared to water. MCAc was also lower after ASB compared to SSB (Figure 2B; *P* = 0.035). PCAc (Figure 2D) was lower after ASB compared to water (*P* = 0.007). The mixed effects model analyses revealed a significant effect for PCAv (Figure 2C; *P* = 0.031) and PETCO_2_ (Table 1; *P* = 0.037), however, no multiple comparisons were significant (*P* > 0.071). There were no differences in mean arterial pressure, stroke volume, cardiac output, or total peripheral resistance between conditions at post-consumption baseline (Table 1).

### 3.1 Dynamic cerebral autoregulation (dCA)

**Figure 3:**
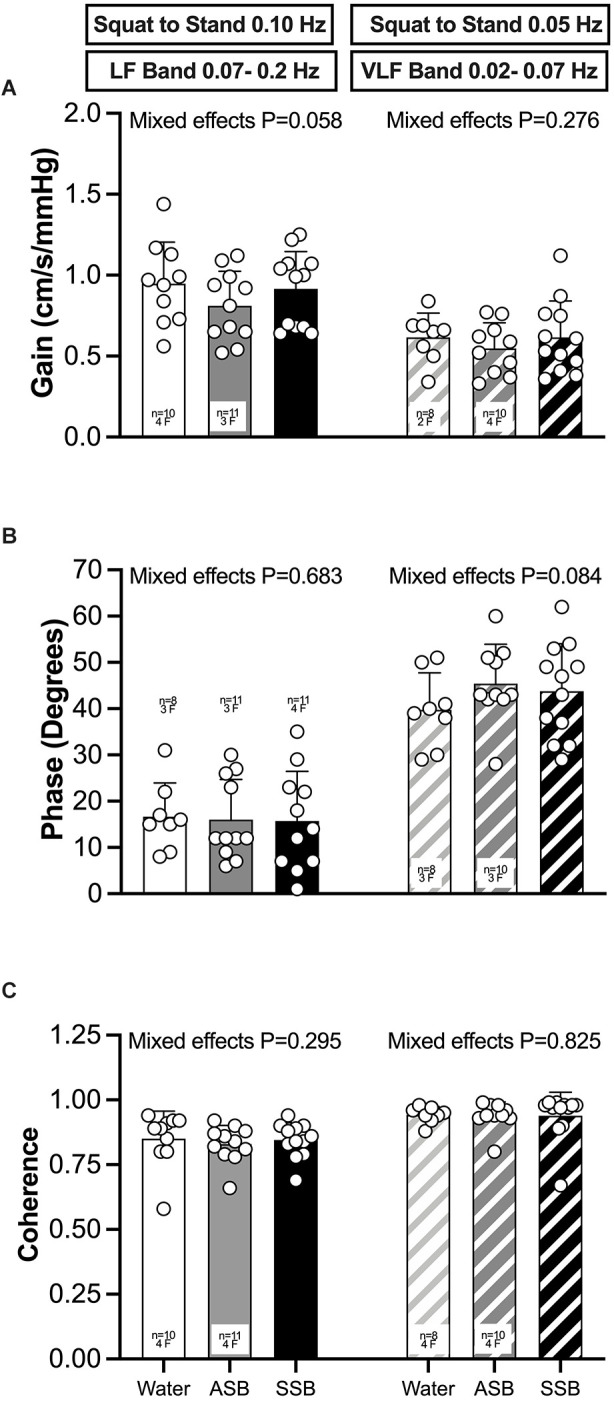
Dynamic cerebral autoregulation mean (bars) ± standard deviation and individual (circles) responses of the middle cerebral artery for gain **(A)**, phase **(B)**, and coherence **(C)** for both squat-to-stand frequencies (0.10 Hz and 0.05 Hz). Mixed effects models were used to compare data between conditions. If a mixed effects model revealed a significant effect, a *post-hoc* Holm-Sidak multiple comparisons test was used to determine where differences existed. Values are presented as the 5 min mean (bars) ± standard deviation and individual responses (circles). *n* = 12, four females unless otherwise noted. Missing data occurred due to exclusion due to noise during transfer function analysis. ASB, artificially-sweetened beverage; SSB, sugar-sweetened beverage.

**Figure 4:**
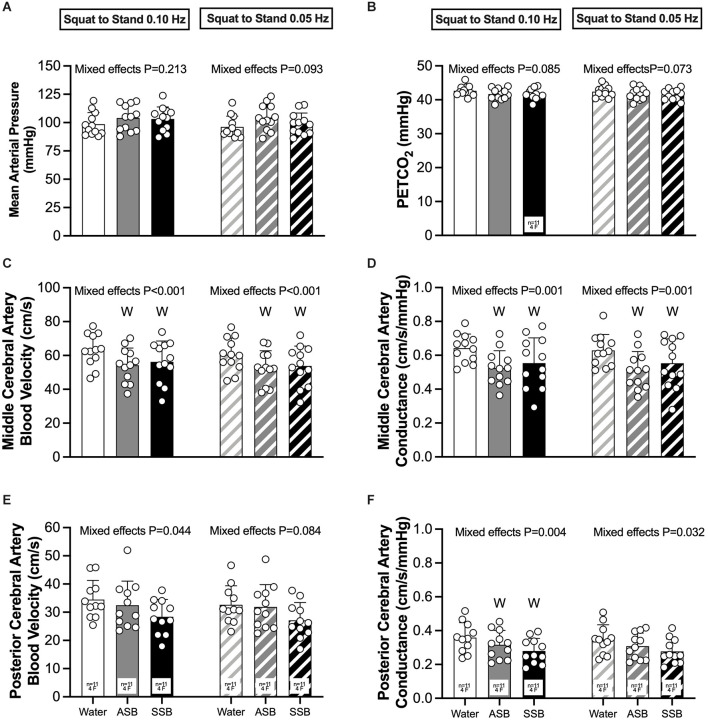
Mean arterial pressure **(A)**, PETCO_2_ **(B)**, middle cerebral artery blood velocity **(C)**, middle cerebral artery conductance **(D)**, posterior cerebral artery blood velocity **(E)**, and posterior cerebral artery conductance **(F)** obtained during each 5 min squat-to-stand frequency (0.10 Hz and 0.05 Hz). Mixed effects models were used to compare data between conditions. If a mixed effects model revealed a significant effect, a *post-hoc* Holm-Sidak multiple comparisons test was used to determine where differences existed. Values are presented as the 5 min mean (bars) ± standard deviation and individual responses (circles). *n* = 12, four females unless otherwise noted. Missing data occurred due to the loss of signal and/or inability to obtain a quality signal while maintaining the same depth and/or gain with the transcranial Doppler signal. ^W^Different from Water (*P* < 0.050); ASB, artificially-sweetened beverage; SSB, sugar-sweetened beverage.

The condition effect for gain, phase and coherence were not different for both squat-to-stand frequencies (*P* ≥ 0.058; Figures 3A–C). The mean responses for mean arterial pressure, PETCO_2_, MCAv, MCAc, PCAv, and PCAc during the 0.10 Hz and 0.05 Hz squat-to-stand protocols are illustrated in Figure 4. The mean cardiovascular responses during the 0.10 Hz and 0.05 Hz squat-to-stand protocols are shown in Table 2. During the 0.10 Hz squat-to-stand frequency, there was no condition effect for MAP (Figure 4A) or PETCO_2_ (Figure 4B). MCAv (Figure 4C) was lower after Diet (54.2 ± 10.2 cm/s) and after Soda (56.2 ± 12.3 cm/s) compared to water (63.2 ± 9.6 cm/s; *P* < 0.001; *P* < 0.001 and *P* = 0.010, respectively). MCAc (Figure 4D) was lower after Diet (0.52 ± 0.10 cm/s/mmHg) and after Soda (0.55 ± 0.15 cm/s/mmHg) compared to water (0.64 ± 0.08 cm/s/mmHg; *P* < 0.001 and *P* = 0.022, respectively). There was a condition effect for PCAv (Figure 4E) but the multiple comparisons did not reveal any significant differences (*P* > 0.061). PCAc (Figure 4F) was lower after Diet (0.32 ± 0.08 cm/s/mmHg) and after Soda (0.28 ± 0.07 cm/s/mmHg) compared to water (0.36 ± 0.9 cm/s/mmHg; *P* = 0.018 and *P* = 0.016, respectively). During the 0.05 Hz squat-to-stand frequency, there was no condition effect for MAP (Figure 4A) or PETCO_2_ (Figure 4B). MCAv (Figure 4C) was lower after Diet (52.9 ± 9.9 cm/s) and Soda (53.8 ± 11.8 cm/s) compared to after water (60.3 ± 9.8 cm/s; *P* < 0.001 and *P* = 0.003, respectively). MCAc (Figure 4D) was lower after Diet (0.51 ± 0.11 cm/s/mmHg) and after Soda (0.55 ± 0.14 cm/s/mmHg) compared to after water (0.63 ± 0.09 cm/s/mmHg; *P* < 0.001 and *P* = 0.042, respectively). There was no condition effect for PCAv (Figure 4E). There was a condition effect for PCAc (Figure 4F) but the multiple comparisons did not reveal any significant differences (*P* > 0.064).

### 3.2 Cerebral vascular reactivity to hypercapnia (CVR_CO2_)

**Figure 5:**
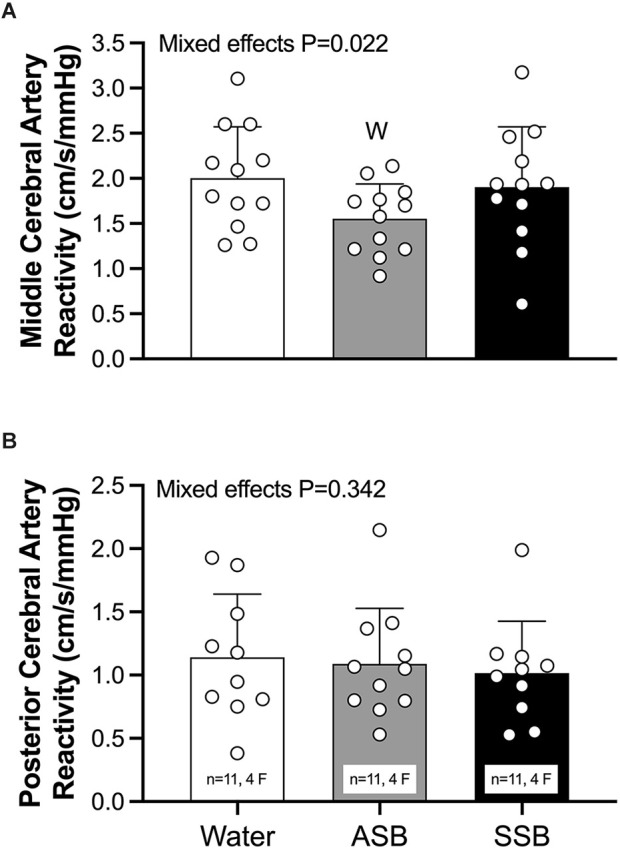
Left middle cerebral artery **(A)** and right posterior cerebral artery **(B)** reactivity to hypercapnia. Mixed effects models were used to compare data between conditions. If a mixed effects model revealed a significant effect, a *post-hoc* Holm-Sidak multiple comparisons test was used to determine where differences existed. Data are presented as mean (bars) ± standard deviation and individual (circles) responses. *n* = 12, four females unless otherwise noted. Missing data occurred due to the loss of signal and/or inability to obtain a quality signal while maintaining the same depth and/or gain with the transcranial Doppler signal. ^W^Different from Water (*P* < 0.050); ASB, artificially-sweetened beverage; SSB, sugar-sweetened beverage.

**Figure 6:**
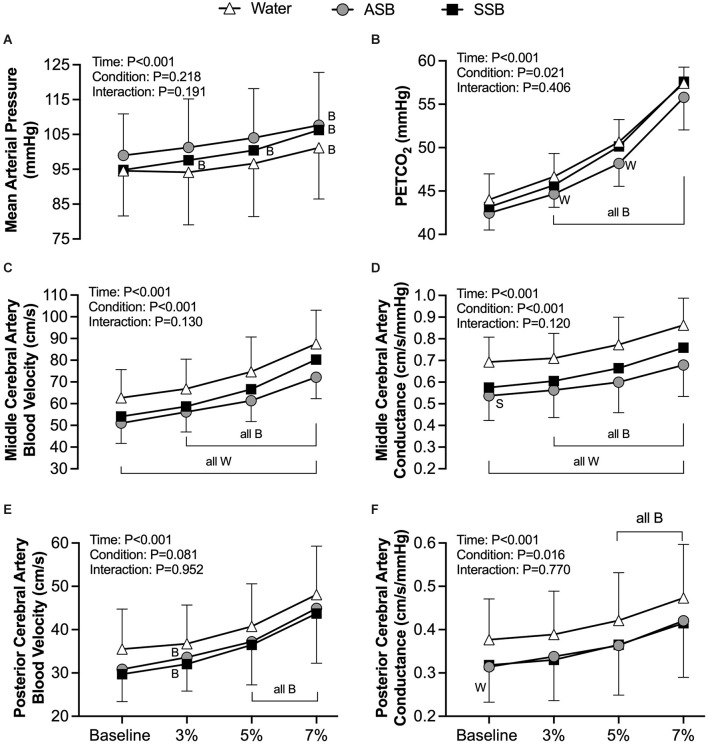
Mean arterial pressure **(A)**, PETCO_2_ **(B)**, middle cerebral artery blood velocity **(C)**, middle cerebral artery conductance **(D)**, posterior cerebral artery blood velocity (**E**; *n* = 10, three females for water, *n* = 11, *n* = 4 for ASB, and *n* = 10, two females for SSB), and posterior cerebral artery conductance (**F**; *n* = 10, three females for water, *n* = 11, four females for ASB, and *n* = 10, two females for SSB) obtained from each stage of the cerebral vascular reactivity to hypercapnia test. Mixed effects models were used to compare data between conditions. If a mixed effects model revealed a significant effect, a *post-hoc* Holm-Sidak multiple comparisons test was used to determine where differences existed. Data are presented as mean ± standard deviation. *n* = 12, four females unless otherwise noted. Missing data occurred due to the loss of signal and/or inability to obtain a quality signal while maintaining the same depth and/or gain with the transcranial Doppler signal. ^B^Different from baseline (*P* < 0.050); ^W^Different from water (*P* < 0.050); ^S^Different between soft drinks (*p* < 0.050); all B = all three beverages are different (*P* < 0.050) from baseline; all W = both the SSB and ASB are different (*P* < 0.050) from water; ASB, artificially-sweetened beverage; SSB, sugar-sweetened beverage.

CVR_CO2_ in the MCA (Figure 5A) was lower after ASB (1.55 ± 0.38 cm/s/mmHg) compared to water (2.00 ± 0.57 cm/s/mmHg; *P* = 0.011) but did not differ between water and SSB (1.90 ± 067 cm/s/mmHg; *P* = 0.593) or between soft drinks (*P* = 0.056). There was no effect of beverage on CVR_CO2_ in the PCA (ANOVA *P* = 0.342; Figure 5B). The mean cardiovascular responses during each stage of the cerebral vascular reactivity test are presented in Table 2. The mean responses for mean arterial pressure, PETCO_2_, MCAv, MCAc, PCAv, and PCAc responses are illustrated in Figure 6. During CVR_CO2_, mean arterial pressure (Figure 6A) was elevated compared to baseline at all three stages with the SSB only and at 7%CO_2_ for all three beverages. PETCO_2_ (Figure 6B) was lower after Diet vs. water during 3% CO_2_ (45 ± 2 vs. 47 ± 3 mmHg; *P* = 0.023) and 5% CO_2_ (48 ± 3 vs. 51 ± 3 mmHg; *P* = 0.047). There were no differences in PETCO_2_ between Soda vs. water (*P* > 0.076) or between soft drinks (*P* > 0.076) during CVR_CO2_. MCAv (Figure 6C) increased from baseline at all stages of CVR_CO2_ after all beverages (all *P* < 0.003). MCAv was lower after Soda vs. water during 3% CO_2_ (58.7 ± 12.8 vs. 66.9 ± 13.6 cm/s; *P* = 0.001), 5% CO_2_ (66.6 ± 15.9 vs. 74.7 ± 16.0 cm/s; *P* = 0.007), and 7% CO_2_ (80.3 ± 18.7 vs. 87.5 ± 15.5 cm/s; *P* = 0.040). MCAv was also lower after Diet vs. water during 3% CO_2_ (56.2 ± 9.3 vs. 66.9 ± 13.6 cm/s; *P* = 0.001), 5% CO_2_ (61.4 ± 9.6 vs. 74.7 ± 16.0 cm/s; *P* = 0.004), and 7% CO_2_ (72.1 ± 87.5 ± 15.5 cm/s; *P* = 0.002). There were no differences in MCAv between soft drinks (*P* > 0.063) during CVR_CO2_. MCAc (Figure 6D) increased from baseline at all stages of CVR_CO2_ after all beverages (all *P* < 0.020). MCAc was lower after Soda vs. water during 3% CO_2_ (0.61 ± 0.14 vs. 0.71 ± 0.12 cm/s/mmHg *P* < 0.001), 5% CO_2_ (0.67 ± 0.17 vs. 0.77 ± 0.13 cm/s/mmHg *P* = 0.003), and 7% CO_2_ (0.76 ± 0.19 vs. 0.86 ± 0.13 cm/s/mmHg *P* = 0.016). MCAc was also lower after Diet vs. water during 3% CO_2_ (0.56 ± 0.13 vs. 0.71 ± 0.12 cm/s/mmHg; *P* < 0.001), 5% CO_2_ (0.60 ± 0.14 vs. 0.77 ± 0.13 cm/s/mmHg *P* = 0.002), and 7% CO_2_ (0.68 ± 0.15 vs. 0.86 ± 13 cm/s/mmHg *P* = 0.001). There were no differences in MCAc between soft drinks (*P* > 0.087) during CVR_CO2_. PCAv (Figure 6E) increased from baseline at all stages of CVR_CO2_ after all beverages (all *P* < 0.004) except for at 3%CO_2_ after water. There was no condition effect for PCAv (*P* = 0.081). PCAc (Figure 6F) increased from baseline at 5% and 7% CO_2_ after all beverages (all *P* < 0.004). There was a condition effect for PCAc but the multiple comparisons did not reveal any significant differences during CVR_CO2_ (*P* > 0.072).

## 4 Discussion

We investigated the acute effects of consuming a caffeinated SSB and ASB on cerebral vascular function in healthy, young adults. The novel findings from our study indicate that compared to water: (1) resting MCAv, MCAc, and PCAc were lower after the caffeinated SSB and ASB; (2) dynamic cerebral autoregulation did not differ with the SSB compared to the ASB or between soft drinks and water during an orthostatic challenge; and (3) cerebral vascular reactivity to hypercapnia did not differ between the SSB and ASB but was attenuated after ASB compared to water.

MCAv was lower following consumption of both soft drink beverages compared to water. Cerebral vascular conductance, an indirect indicator of vasomotor tone (Claassen et al., 2007), was also lower in the MCA and PCA following both soft drinks compared to water. We also observed a significant effect of beverage consumption on PCAv, but the *post hoc* analysis was unable to determine differences among the beverages. Although unlikely, the lower MCAv, MCAc, and PCAc values could be a result of small changes in arterial CO_2_ content as mean arterial pressure was not different between conditions. Cerebral artery blood velocity is sensitive to changes in arterial CO_2_ content such that there is an estimated 2% decrease in velocity per mmHg decline in PETCO_2_ (Ide et al., 2003; Hoiland et al., 2019). Although not significant, the changes in PETCO_2_ compared to water were ~2 mmHg and ~1 mmHg lower following ASB and SSB, respectively. These numbers are similar to the non-significant ~2 mmHg difference in PETCO_2_ after 300 mg of caffeine, however, our caffeine content (76 mg) was much lower than this (Blaha et al., 2007). Thus, it is unlikely that these small differences in arterial CO_2_ content explain the entire magnitude of the lower MCAv following ASB (~18%) and SSB (~14%) compared to water. Both soft drinks listed identical amounts of caffeine (76 mg), which is an adenosine receptor antagonist that peaks in the plasma between 30 and 90 min after consumption (Mandel, 2002; Martins et al., 2020) and causes vasoconstriction of the cerebral the vasculature, thus reducing cerebral blood flow (Cameron et al., 1990; Coney and Marshall, 1998; Phillis and O’Regan, 2003). For example, 250 mg (Lunt et al., 2004) or 300 mg (Blaha et al., 2007) of caffeine ingestion reduced MCAv by 13% and 14%, respectively, in both adults who were healthy and post-stroke. It is currently not known if a dose-response relationship exists between caffeine ingestion and intracranial artery blood velocity. Nonetheless, the caffeine content in the ASB and SSB likely contributed to the lower resting intracranial artery blood velocities and indices of vasodilation (i.e., conductance) that we observed compared to water. Although we did not collect blood samples in this study, we tested participants within the window of peak plasma caffeine concentrations (Mandel, 2002; Martins et al., 2020).

In addition to caffeine, other constituents of the ASB and SSB may have modulated resting intracranial artery blood velocities and indices of vasodilation (i.e., conductance) that we observed compared to water. The ASB that we used contained the artificial sweeteners acesulfame potassium, aspartame, and sucralose. There is currently no evidence in humans to indicate that the acute consumption of these artificial sweeteners would attenuate resting cerebral blood flow. However, there is evidence that ingesting acesulfame potassium combined with sucralose for 10 weeks reduces endothelium-dependent relaxation to acetylcholine in rat aortas (Risdon et al., 2020). Therefore, chronic consumption of acesulfame potassium and sucralose may be needed before observing changes in resting intracranial cerebral artery blood velocity.

Acute consumption of an HFCS beverage increased renal vascular resistance (Chapman et al., 2020) and increased systolic blood pressure (Le et al., 2012) but did not affect arterial stiffness in young healthy adults (Freemas et al., 2021). Although we do not have pre-experimental beverage consumption blood pressure, there were no differences between the blood pressures at baseline between water and soft drink beverages but this could be due to a lower amount of caffeine than amounts known (>90 mg) to increase blood pressure (Nurminen et al., 1999). To our knowledge, there are no reports on the effects of HFCS on cerebral blood velocity and cerebral vascular function. However, there are reports of the isolated acute consumption of fructose. The acute ingestion of fructose mixed with water has been shown to reduce cerebral blood flow, assessed using magnetic resonance imaging arterial spin labeling, to various brain regions such as the thalamus, hippocampus, posterior cingulate cortex, fusiform gyrus, and visual cortex (Page et al., 2013). The acute consumption of fructose also reduced serum biomarkers of nitric oxide, increased serum uric acid, and impaired micro- and macrovascular endothelial function in healthy adults (Loader et al., 2017; Cai et al., 2018). Fructose-fed rats showed increased levels of oxidative stress after 7 days of consumption (Delbosc et al., 2005). There is also evidence that fructose ingestion modulates sympathetically-mediated increases in heart rate (Schwarz et al., 1992). Our findings likely support this as heart rate was higher at post-consumption baseline with the SSB compared to water and ASB, despite having the same caffeine amount as the ASB. Together, it is important to consider the route of administration, interaction with other ingredients in the drink (e.g., caffeine), the volume of fructose, the timing of measurements, etc. as these could influence modulators of cerebral vascular function similar to the peripheral vasculature.

We found that CVR_CO2_ in the MCA was lower following the ingestion of ASB compared to water but observed no other differences between beverages or in the PCA. Using an animal model, relatively high doses of caffeine (10 mg/kg) have been shown to reduce the cerebral vascular response to hypercapnia by 40–44% (Estevez and Phillis, 1997). Using transcranial Doppler ultrasound, Blaha et al. (2007) also found that ingestion of 250 mg of caffeine reduced the MCAv response to hypercapnia by ~11%. Conversely, the intravenous infusion of 2.5 mg/kg body weight of caffeine did not reduce the cerebral blood flow response to hypercapnia assessed using blood-oxygenation-level-dependent magnetic resonance imaging (Chen and Parrish, 2009). Because we only observed a decrease in CVR_CO2_ following ASB, even though both soft drinks contained identical caffeine content, we speculate that additional factors modulated this response. CVR_CO2_ is used as an index of cerebral vasculature nitric oxide bioavailability as it facilitates increases in MCAv during hypercapnia (Iadecola and Zhang, 1994; Schmetterer et al., 1997). However, our study is the first to examine such a response following ASB. In this regard, flow-mediated dilation (a marker of peripheral vascular nitric oxide bioavailability; Joannides et al., 1995) was found to be not meaningfully different following the acute consumption of sucralose in healthy, young adults (Memon et al., 2014). Increases in cerebral blood flow during hypercapnia are also due to the hyperpolarization of endothelial cells *via* the activation of voltage-gated potassium channels resulting in vascular relaxation (Kitazono et al., 1995; Nelson and Quayle, 1995) which can be assessed with selective inhibitors, such as glibenclamide (Faraci and Heistad, 1998). Additional studies are warranted to replicate our results and determine which constituents of ASB contribute to the attenuated CVR_CO2_.

Although we did not collect blood to quantify blood biomarkers such as uric acid, nitric oxide precursors, blood glucose and fructose, insulin, and lipids, we have previously shown that HFCS ingestion increases serum uric acid, serum glucose, and serum fructose following the identical SSB used in the current study (Chapman et al., 2020). Uric acid has been linked with reducing nitric oxide bioavailability (Khosla et al., 2005). Thus, we expected CVR_CO2_ to be attenuated following the SSB. Our study is the first to report whether the consumption of SSB influences CVR_CO2_, therefore it is unclear why CVR_CO2_ was not affected by SSB ingestion. We speculate that the amount of circulating uric acid was not sufficient to lower nitric oxide bioavailability and influence CVR_CO2_ in the intracranial arteries we examined. Furthermore, fructose consumption does cause small increases in circulating insulin (Le et al., 2012), which might have offset the vasoconstricting effects of uric acid and caffeine in the cerebral vasculature during hypercapnia (Katakam et al., 2009). Without blood samples, we are unable to identify blood biomarkers that could have helped elucidate specific mechanisms.

We found that dynamic cerebral autoregulation during an orthostatic stressor was not statistically different between the beverage conditions. Our study is the first to report how ASB and SSB influence dynamic cerebral autoregulation in humans. Moderate and large doses of caffeine (20 and 40 mg/kg body weight) administered to rats did not alter cerebral autoregulation during severe hypotension (Phillis and DeLong, 1986). Previous evidence from humans indicates that the rate of regulation, a marker of dynamic cerebral autoregulation, is greater (i.e., improved) 30 min following acute coffee ingestion that included 250 mg of caffeine among other ingredients that might contribute to augmented cerebrovascular function (Sasaki et al., 2016). Sasaki et al. (2016) posited that caffeine ingestion caused cerebral vasoconstriction, due to increased circulating catecholamines and antagonizing cerebral adenosine receptors, that lead to augmented dynamic cerebral autoregulation. We observed lower resting MCAc and PCAc following ASB compared to water indicating intracranial vasoconstriction. However, these changes in resting cerebral vasomotor tone did not translate to an augmented dynamic cerebral autoregulation. Thus, it is possible that caffeine content greater than our ASB and SSB or other drink constituents of the ASB and SSB that we tested is needed to influence cerebral vasoconstriction to the extent needed to influence dynamic cerebral autoregulation in humans during orthostatic stress.

### 4.1 Experimental considerations

There are several experimental considerations that should be considered when interpreting our data. First, a technological limitation of transcranial Doppler ultrasound of intracranial cerebral arteries is the inability to measure the arterial diameter. Thus, we are assuming that the diameter of these arteries remained constant and any changes in velocity were due to changes in blood flow (Willie et al., 2011). That being said, Lunt et al. (2004) suggested that vasoconstriction occurs in the MCA following caffeine consumption. Second, we did not measure blood flow in extracranial cerebral arteries such as the internal carotid and/or vertebral artery that could have provided additional information regarding global cerebral blood flow. Third and as mentioned above, we did not collect blood samples to quantify serum changes in caffeine, blood glucose, insulin, lipids, uric acid, or nitric oxide precursors. Therefore, it is unclear if these modulators of arterial function were different between beverage conditions. Our laboratory previously found that serum uric acid was greater following SSB consumption compared to ASB and water, serum glucose increased with the ASB and SSB compared to water, and serum fructose increased with the SSB compared to water using the same beverages and similar timing of measurements as our current study (Chapman et al., 2020). Fourth, we used young participants who self-reported to be healthy. Therefore, we cannot extend our findings to older and/or patient populations that have cerebral vascular risk factors. Fifth, we recruited both males and females to participate in our study but we were not statistically powered to make sex comparisons as this was not an objective of our study. Although we did schedule female participants within the same phase of the menstrual cycle, we also could not examine responses throughout the menstrual cycle. Sixth, we did not collect food and beverage dietary logs nor did we provide a standardized meal prior to each study visit. The restriction of food for at least 2 h prior to each study visit may not have been adequate enough to prevent confounding effects of food and/or beverages consumed prior to the study. Finally, our control condition was water. A time control condition without any beverage consumption or completing the cerebral vascular function testing before and after each beverage within the same study visit would have provided additional information regarding the consumption of fluid on cerebral vascular function.

### 4.2 Perspectives and significance

Many individuals take it upon themselves, or they are recommended by a health care provider, to limit fructose intake and/or reduce caloric intake for a variety of reasons that may include reducing cardiovascular and metabolic disease risks. Despite these good intentions to limit caloric and fructose intake, stroke risk is still elevated in people that regularly consume ASB compared to individuals who do not (Bernstein et al., 2012; Pase et al., 2017). Thus, we investigated how the acute consumption of SSB and ASB influence cerebral vascular function. Our results indicate intracranial cerebral vasoconstriction following ASB and SSB and a lower cerebral vasomotor response to hypercapnia following ASB compared to water. We speculate that caffeine is the main ingredient that contributed to these changes in cerebral vascular function. However, stroke risk (Larsson and Orsini, 2011) and all-cause mortality (Crippa et al., 2014) in regular coffee drinkers is lower than non-coffee drinkers. Therefore, although caffeine was likely the main contributor to the acute changes we observed, other ingredients in soft drinks may contribute to the associated negative health outcomes with repeated SSB and ASB consumption. Proper cerebral vascular function during orthostatic challenges, such as a squat-to-stand protocol, is essential for the maintenance of cerebral blood flow and the prevention of syncope.

## 5 Conclusions

Our study is the first investigation to report on the acute effects of caffeinated-soft drinks, both sugar- and artificially-sweetened, on cerebral vascular function. Our findings demonstrate: (1) lower blood velocity and conductance in the middle cerebral artery following an artificially sweetened beverage and a sugar-sweetened beverage and lower posterior cerebral artery conductance following an artificially sweetened beverage compared to water; and (2) lower cerebral vascular reactivity to hypercapnia in the middle cerebral artery following an artificially sweetened beverage compared to water. Contrary to our hypothesis, we did not observe any differences between water consumption and a sugar-sweetened beverage for cerebral vascular reactivity to hypercapnia or any differences between beverage conditions for dynamic cerebral autoregulation. We speculate that caffeine is the main ingredient that modulated resting intracranial artery blood velocity and cerebral vascular reactivity to hypercapnia following caffeinated beverages.

## Data Availability Statement

The raw data supporting the conclusions of this article will be made available by the authors, without undue reservation.

## Ethics Statement

The studies involving human participants were reviewed and approved by Institutional Review Board of the University at Buffalo. The patients/participants provided their written informed consent to participate in this study.

## Author Contributions

ER, MW, ZS, and BJ: study design. ER, MW, PK, and LP: collected data. ER, MW, PK, LP, and BJ: analyzed data. ER, MW, PK, and BJ: drafted manuscript. ER, MW, PK, LP, ZS, and BJ: edited and approved the final submission. All authors contributed to the article and approved the submitted version.
